# Creation and Implementation of an Electronic Sexual Assault Record at the Geneva University Hospital

**DOI:** 10.2196/66764

**Published:** 2025-11-20

**Authors:** Sara Cottler-Casanova, Laurène Rimondi, Monique Lamuela Naulin, Tony Fracasso, Jasmine Abdulcadir

**Affiliations:** 1 Division of Gynecology, Department of Woman, Child and Adolescent, Geneva University Hospitals, Bd de la Cluse 30, Geneva, 1205, Switzerland, 41 765526978, 41 7955350; 2 Faculty of Medicine, University of Geneva, Geneva, Switzerland; 3 Centre universitaire romand de médecine légale, Geneva, Lausanne, Switzerland

**Keywords:** Geneva, sexual assault, data collection, dataset, electronic health records, registry, administrative hospital data, computerized medical records, medical records, EHR, health records, records, hospital data, Europe, European, Obstetrics, Gynecology, EMR, efficiency, clinical workflow, Swiss, Switzerland, implementation, administrative

## Abstract

**Background:**

In Switzerland, sexual assault reports have historically been documented on paper, which limited standardization, completeness, and challenges to produce reliable statistics.

**Objective:**

This study describes the development and implementation of an Electronic Sexual Assault Record (eSAR) within Geneva University Hospitals’ Electronic Medical Record (EMR) system, with the aim of improving data quality, documentation, and multidisciplinary coordination.

**Methods:**

The eSAR was developed by a multidisciplinary team including forensic doctors, gynecologists, nurses (clinical and informatics), epidemiologists, and IT specialists. Its structure was based on existing hospital protocols and international recommendations. Variables were defined as “essential” or “highly recommended,” with structured fields to ensure completeness and comparability. Confidentiality was safeguarded through restricted access and regular audits.

**Results:**

The eSAR was launched in June 2022 and revised in 2023 after user feedback and training. Since implementation, 382 reports have been completed. Data quality improved substantially, with major reductions in missing information. The system also streamlined workflows and strengthened collaboration across specialties.

**Conclusions:**

The eSAR improved documentation and data reliability, providing a replicable model for standardized sexual assault reporting in Switzerland.

## Introduction

### The Burden of Sexual Violence in Switzerland

Sexual violence is a serious public health concern in Switzerland. According to a 2019 population-based survey involving 4500 women, 22% of women and girls aged 16 and older reported having experienced nonconsensual sexual acts, and 12% had sex against their will [1]. In 2023, police and victim assistance centers (LAVI/Opferhilfe) reported a total of 1647 cases of sexual violence against women to the Swiss Federal Statistical Office, including 822 cases of rape (Art. 190) and 549 cases of sexual assault (Art. 189) [2].

Switzerland ratified the Istanbul Convention in 2017. Since then, monitoring efforts by the Council of Europe’s Group of Experts on Action against Violence against Women and Domestic Violence (GREVIO) have highlighted several gaps and issued specific recommendations. These include strengthening standardized postrape care pathways, such as screening, treatment, injury documentation, and referral to specialized services; improving health care professional training across disciplines, and establishing harmonized standards for forensic data collection throughout the country [3].

To support implementation of these recommendations, the Swiss National Action Plan (PAN CI) [4] for 2022‐2026 was adopted. It prioritized three urgent measures: (1) scaling up post-rape care services across cantons (2) developing harmonized healthcare protocols, and (3) improving national statistics to support evidence-based policymaking.

In Romandie, the French-speaking part of Switzerland, several cantons, including Geneva, Vaud and Valais have established specialized sexual assault support centers that reflect GREVIO’s standards. These cantons offer joint forensic and gynecological examinations within emergency settings, conducted by medical professionals trained in trauma-informed care. Importantly, access to these services is not contingent on filing a police report, reinforcing a survivor-centered approach to care. Treatment includes postexposure prophylaxis for HIV and sexually transmitted infections (STIs), emergency contraception, crisis mental health support, and referrals for medium- and long-term follow-up [5]. Patients may be directed to a range of specialties within the hospital network, including infectious diseases, psychiatry, the Interdisciplinary Unit for Medicine and Prevention of Violence (UIMPV), addiction medicine, and surgical specialties when needed [5,6].

In parallel to providing clinical care, medico-legal exams serve the function of collecting and documenting forensic evidence. Accurate and thorough documents can improve judicial outcomes by increasing the likelihood of successful prosecution [7]. However, because there is not unified national protocol for the medico-legal exam, data collection practices vary across institutions. The development of harmonized cantonal and national guidelines depends on the availability of high-quality, systematically collected data.

### Limitations of Manual Documentation

Until 2021, medico-legal sexual assault reports (SARs) at the Valais, Vaud and Geneva centers were completed by hand. While this method supported a detailed narrative documentation, it posed several challenges. A retrospective study conducted between 2018 and 2021 on 740 sexual assault reports from Geneva and Lausanne University Hospitals revealed areas for improvement, particularly regarding the consistency and comprehensiveness of forensic documentation [8]. Accurate and thorough documentation is crucial not only for optimal patient care but also for judicial decision-making, including whether cases proceed to trial [9].

Despite the HUG’s (Geneva University Hospital) reputation as a standard for trauma-informed care, the manual completion of sexual assault reports across multiple departments contributed to administrative delays and occasional data gaps. This variability in documentation impacted the ability to systematically capture essential information such as assault context, risk factors, and sociodemographic characteristics of both victims and perpetrators. Whether or not the patient had previously experienced sexual violence was not documented in 64% of sexual assault records. Between 34% and 50% of all sexual assault reports were missing information on treatments that were prescribed (eg, emergency contraception, gonorrhea, chlamydia, HIV prophylaxis).

### The Need for a Digital Standardized Report

The absence of a standardized and harmonized approach to data collection posed challenges in evaluating service utilization and identifying unmet needs.

Consequently, the ability to generate evidence-based recommendations for strengthening postrape care services, in line with public health strategies at a national and international level such as those from the Istanbul Convention and GREVIO, were limited.

### Aims and Objectives

Recognizing these opportunities for improvement, HUG initiated the development of an Electronic Sexual Assault Record (eSAR) within its Electronic Medical Record (EMR) system. This innovation aimed to maintain HUG’s standard of care while enhancing data quality, streamlining clinical workflows, and supporting multidisciplinary collaboration. Our primary aim is to describe the eSAR and its creation and implementation into the HUG’s existing EMR System. The report is structured following the iCHECK-DH (Guidelines and Checklist for the Reporting on Digital Health Implementations) guidelines [10].

## Methods

### Technical Design

The integration of the eSAR into the EMR system was strategically planned from the outset, following the findings of the 2022 retrospective report as the optimal solution for reducing inconsistencies, improving data completeness, and providing high-quality evidence to inform policy-making and the development of best-practice guidelines. The Chiefs of Forensic Medicine and the Gynecology and Obstetrics Emergency Department proactively engaged with the hospital’s medical leadership and the information systems directorate to initiate the creation of the eSAR. A nurse specialized in informatics was allocated to the project to help with the development of the digital form within the EMR system. The design of the eSAR involved a multidisciplinary team, including nurse specialized in informatics, a nurse from the Gynecology and Obstetrics Emergency Department, an epidemiologist, a statistician, the Chief of the Gynecology and Obstetrics Emergency Department, and the Chief of the Forensic Medicine unit.

The eSAR was also multidisciplinary in its design. Given that gynecologists and forensic doctors from two separate departments needed access to create, modify, and sign the same record, the informatics directorate developed a specialized protocol granting multiple units access to the shared form. The eSAR workflow is fully integrated across both units, and for traceability, all user interactions, including who accessed the form and what modifications were made, are systematically recorded.

### Target

The eSAR was developed to be used by forensic doctors, gynecologists and nurses for all sexual assault reports made in the Gynecology and Obstetrics Emergency Department at the HUG. The eSAR needed to be valid for any patient seen in this emergency unit including cisgender women, nonbinary, queer, transgender men with a vulva and vagina and transgender women- regardless of age. While the patients under 16 are treated in the pediatric department, the gynecological exam is conducted in the Gynecology and Obstetrics Emergency Department. The eSAR may also be used in the gynecology outpatient clinic, where sexual assault reports are conducted if more than 7 days has elapsed since the sexual assault. Past seven days, evidence on the patient’s body will most likely have disappeared and the intervention of a forensic doctor is no longer necessary. The eSAR is also used, regardless of whether the medico-legal exam was mandated by the police or the public prosecutor.

The multidisciplinary team synthesized input from clinical practice guidelines, expert clinical knowledge, and relevant classifications, international recommendations, and guidelines. The starting point was HUG’s annually revised clinical practice guidelines for documenting sexual assault, known as “attitude sheets” [11]. Additional sources included international recommendations and clinical scales, such as World Health Organization’s guidelines [12-14], the International Classification of Diseases, the Tanner Score, European AIDS Clinical Society recommendations [15], and ethical considerations on gender from the Inclusion Group of Geneva University [16]. This comprehensive approach ensured that the eSAR was evidence-based, aligned with best practices, and tailored to meet the needs of both healthcare providers and patients.

The preliminary sexual assault report form framework included socio-demographic and clinical characteristics, risk factors, complaint status, relationship to the perpetrator, description of events, genital, anal and bodily injuries, pictures, and a description of samples collected. Once the overall structure was outlined, the team designed the individual variables using binary options, multiple-choice selections, and drop-down lists. Most fields are structured to ensure the desired information is captured, in a neutral and unbiased way. Free-text fields were retained as additional fields, as requested by the forensic doctors to ensure that all information could be documented. However, the structured fields ensure consistency, comparability and ease of analysis for research purposes.

3 key performance indicators were envisaged to evaluate the eSAR:

- Descriptive statistics of the number of eSARs in progress and finalized
- Proportion of eSARs with no missing data
- Access to the eSARs by authorized health care professionals only and verifiable

### Blueprint Summary

The sexual assault report framework is composed of eight sections (Table 1).

**Table 1.** The 8 sections composing the sexual assault report framework.

Section	Description
Sexual assault characteristics	Details on sexual assault, including: (1) patient characteristics, (2) assault characteristics, including (3) perpetrator characteristics, and (4) violence characteristics
Post-Assault Characteristics	Details on post-assault medical and forensic evaluations, including: (1) forensic exam and evidence, (2) gynecological exam, and (3) service referrals and treatment
Forensic Laboratory Kit	Comprehensive list of samples collected, including swabs, smears, slides, blood, urine, and saliva. It documents the date, time, and signature of the forensic doctor
Infectious Diseases Consultation Request Form	Completed when Post-Exposure Prophylaxis (PEP) for HIV prevention is administered and a consultation with the infectious disease department is requested
Confidentiality/Release of Information Form	Signed by the patient to authorize the release of information to police and magistrates if a complaint is filed; a hand-signed copy is scanned for the record
Employment Leave Certificate	Assesses work capacity and the possibility of filing an accident report for health or accident insurance, in compliance with Swiss law
Provisional Medical Certificate	Printed for the patient and scanned for the record. It provides initial findings, a summary of the medical consultation, additional remarks, and signatures of the gynecologist and forensic physician
Full eSAR^a^ Patient Information Document	Completed by the gynecologist and forensic physician, handed to the patient. It includes a summary of medical examinations, test results, treatments administered (eg, emergency contraception, antibiotics, HIV prophylaxis), follow-up appointments, and documents provided to the patient

a e-SAR: electronic sexual assault records.

### Data

The variables were divided in two categories: “essential” variables that are required to sign the file, and “highly recommended” variables, to provide more details depending on medico-legal situation. A pop-up window indicates whether any data is missing; both essential and highly recommended. To ensure harmonized data entry, the users can consult definitions and detailed descriptions of lesion types and diagrams of anatomical sites. The exact anatomical location and terminology for genital injuries are provided in a diagram of the vulva. An automatic data quality check was built into the system to identify any data entry errors, such as extreme outliers.

The results of the laboratory tests for pregnancy and STIs, as blood chemistry, complete blood count and vaginal bacteriology, are available in the patient’s EMR. The monthly list of health professionals accessing the eSAR are reviewed by the CURML (Centre Universitaire Romand de Médecine Légale, University Center for Legal Medicine in French-speaking Switzerland) to verify the access of only authorized health professionals. The triage nurse collects blood and urine samples for toxicological analysis, and ensures their dispatch to the laboratory or Medico-Legal Institute. During the medical examination, the nurse also coordinates follow-up appointments and administers necessary prophylactic treatments. Employment leave and medical certificates are printed and an alert is sent to the laboratory to warn about the arrival of the samples for STIs, improving traceability.

### Confidentiality

To open the eSAR, the user must be authorized and provide justification. Access rights are restricted to gynecologists, forensic doctors and nurses directly involved in the medical care of the patient’s sexual assault, together with the infectious disease specialists, the psychologists and medical team of the UIMPV and other medical staff (eg, proctologist, psychiatrist, pediatrician, otorhinolaryngologist). Strict confidentiality measures are required to prevent unauthorized access, with any breach punishable by law. To reinforce oversight, a monthly audit report listing all healthcare professionals who accessed the eSAR is reviewed by the CURML. Data security is managed by the IT department of HUG, with all records securely stored on proprietary servers. For research purposes, deidentified data extracts can be obtained through the Magellan informatics module. However, the use of eSAR data for research requires both patient consent and approval from the Swiss ethics committee, ensuring compliance with ethical and legal standards.

## Implementation (Results)

### Content Validation

The content validation process for the eSAR was multi-phased and collaborative. Initially, structured meetings and iterative refinements were conducted by a multidisciplinary team composed of forensic doctors, gynecologists, a nurse from the gynecology and obstetrics emergency department, a nurse specialized in informatics and an epidemiologist. During these early stages, the team reviewed anonymized sexual assault files and implemented several key changes, including standardized terminology (eg, shifting from “victim” to “patient”) (Multimedia Appendix 1), revising question phrasing for clarity, and introducing new variables to better capture details on assault characteristics, injuries, and treatments. Response formats were adjusted, for example by replacing ambiguous free-text responses with binary or multiple-choice options to improve data quality and comparability. The sequence of form sections was also reorganized for greater usability and workflow integration.

The draft eSAR was then presented to clinicians from the Obstetrics and Gynecology Emergency Department for further feedback. Because gynecologists and nurses were already familiar with the hospital’s electronic medical record system, dedicated training sessions were organized specifically for forensic doctors to ensure smooth adoption. The eSAR was officially launched in June 2022 (Table 2).

**Table 2.** The eSAR timeline.

eSAR timeline	Content
2018‐2021	Retrospective study reveals documentation gaps (paper SAR)^a^
Q3/4 2021	Project planning, leadership engagement
Q1 2022	Multidisciplinary team formed, prototype design
Q2 2022	Structured meetings, iterative development, early validation
Q2–Q3 2022	Focus groups, pilot testing, refinement
June 2022	eSAR version 1 launched (Gynecology/ObGyn ED)^b^
Q4 2022	Ongoing user feedback, additional training
Sep 2023	Major revision (version 2; expert feedback, improved flow, new response options)
2023‐2024	Expansion, routine use, continuous updates
2024	Full integration, data extraction or reporting

a SAR: sexual assault report.

b ObGyn ED: Obstetrics and Gynecology Emergency Department.

After implementation of the first eSAR version, expert focus groups were convened to assess usability, clarify clinical workflow, and refine the eSAR structure based on real patient cases. These focus groups comprised 12 participants: 4 forensic doctors, 3 gynecologists, 2 nurses, 2 informatics specialists, and 1 epidemiologist. Discussions in these focus groups mapped out the patient journey during sexual assault consultations, defined clear roles and responsibilities at each care stage, and identified opportunities to streamline data entry while minimizing patient burden.

The most significant set of revisions followed these focus groups and expert feedback, culminating in the release of eSAR version 2 in September 2023. This update included improved question phrasing and form flow, a shift to more neutral terminology, and the addition of a “+” button for documenting multiple injuries with automatic body region classification. New response options such as “does not remember” (for cases of amnesia) and “does not know” (for patients unable to answer) were also introduced, ensuring greater accuracy and completeness.

### Workflow

The focus groups played a key role in clarifying the workflow, particularly in determining how the eSAR is filled out and by whom. These discussions helped refine the division of tasks, ensuring a structured and efficient process. The nurse selects a triage motive according to the Swiss Triage Scale, with the reason for the encounter recorded in the DPI (Dossier Patient Intégré or Integrated Patient File) system and displayed on department screens.

Further, the focus groups provided valuable insights into the data entry process, particularly regarding computer use. Physicians noted that they take turns entering information to maintain patient engagement. For example, the gynecologist switches to the computer while the forensic doctor conducts the medico-legal history, documents bodily traumatic lesions, and collects forensic samples. During the consultation, the gynecologist conducts the exam and administers prophylactic treatments, while the forensic doctor simultaneously completes the electronic record. This streamlined approach minimizes consultation time while ensuring double verification of data, enhancing accuracy and completeness in medical and forensic reporting.

### Results

The digitalization of the sexual assault report has significantly improved data reliability and accessibility, enabling the extraction of accurate statistics with ease. With a single click, data can be retrieved based on the number of eSARs per year, month, or type (legal mandate) providing a clearer picture of trends and service utilization (Tables3  4). Since the eSAR was introduced in June 2022, a total of 382 reports have been recorded, with 134 in 2023 and 162 in 2024, reflecting an increase in the number of sexual assaults reported. Most eSARs (84/134, 63%; 102/162, 63%) were completed without a legal mandate, while 37% (50/134 in 2023 and 60/162 in 2024) were mandated cases. The monthly distribution of eSARs varied, with the highest numbers recorded in July of both 2023 and 2024 (15/134, 12% in 2023 and 19/162, 12% in 2024). The peaks in reported cases in July 2023 and 2024 may be explained by increased summer festivals, holidays, and public awareness campaigns, which together create more risk situations and encourage more survivors to seek care [8].

**Table 3.** Number of eSARs^a^ per year.

eSAR	eSARs (N=382), n	With legal mandate, n (%)	Without legal mandate n, (%)
2022 (01.06.22‐31.12.22)	86	N/A^b^	N/A
2023	134	50 (37)	60 (37)
2024	162	84 (63)	102 (63)

a eSAR: electronic sexual assault records.

b Not applicable.

**Table 4.** Distribution by month of eSARs^a^ (2023).

Month of the eSAR	2023 (n=134), n (%)	2024 (n=162), n (%)
January	5 (4)	16 (10)
February	12 (9)	17 (11)
March	15 (11)	13 (8)
April	12 (9)	11 (7)
May	13 (10)	14 (9)
June	14 (10)	14 (9)
July	15 (12)	19 (12)
August	9 (7)	14 (9)
September	14 (10)	9 (6)
October	10 (7)	17 (11)
November	8 (6)	7 (4)
December	7 (5)	11 (7)

a e-SAR: electronic sexual assault records.

The eSAR is comprised of approximately 500 data points, with 44 “essential variables” covering victim, assault, and violence characteristics, as well as postassault details, forensic evidence, gynecological exams, and service referrals, ensuring that all fundamental aspects of every case are recorded. Additionally, 108 highly recommended data points provide more detailed insights depending on the medico-legal context, including documentation of ano-genital injuries, bodily injuries, and ejaculation. Reliable informatic data extraction is now feasible for clinical purposes and annual reports. Moreover, eSAR data is being used to populate the electronic case report form for a prospective study on sexual assault conducted at HUG, which began in November 2022.

Since its implementation, the eSAR has significantly improved the completeness of essential data documentation compared to the previous paper-based SAR, where missing information posed major challenges. In the earlier system, 64% (n=467) of records lacked information on prior sexual assault history, 75% omitted details about decisions to press charges, and 34% to 50% were missing data on prescribed treatments. With the eSAR, these gaps have been substantially reduced: missing data now account for only 8.5% (n=13, see Table 5 below) regarding prior assault history, 0% (n=0) for whether charges were filed, 32% (n=52) for whether future charges will be filed, and just 3.7% (n=6) for postexposure prophylaxis (PEP).

**Table 5.** Improvements in missing data.

Variables	No, n (%)	Yes, n (%)	Unknown/Missing, n (%)	Not applicable, n
Retrospective study (Before eSAR), n=727	121 (17)	139 (19)	467 (64)	13
2024 (After eSAR implementation), n=162	50 (32.7)	90 (58.8)	13 (8.5)	9

The eSAR also facilitates the medical follow-up of the patient. Patient appointments can be made at the time of the medico-legal consultation with the click of a button. Previously, a fax system was used to send patient appointment information between the ObGyn (Obstetrics and Gynecology) emergency unit and the infectious diseases unit. The eSAR also helps with multidisciplinary medical follow-by infectious disease specialists, as well as the staff of the UIMPV and gynecology outpatient clinic, allowing the doctors and psychologists to read the patient’s sexual assault file and better prepare, without the patient having to repeat details from their assault.

### Efficiency

A crucial outcome for patients is the time needed to finalize the medico-legal report with all the necessary information. The development and the implementation of the eSAR facilitates and streamlines the entire workflow. The administrative burden has been considerably lightened since paper documents are not physically transported anymore through medical departments. The electronic traceability also allows to monitor the administrative process of filling, finalizing and signing the eSAR. The training of new doctors confirmed the implementation of the eSAR facilitates the continuity of the medical care and clinical practice with less experienced doctors.

**Figure 1.**
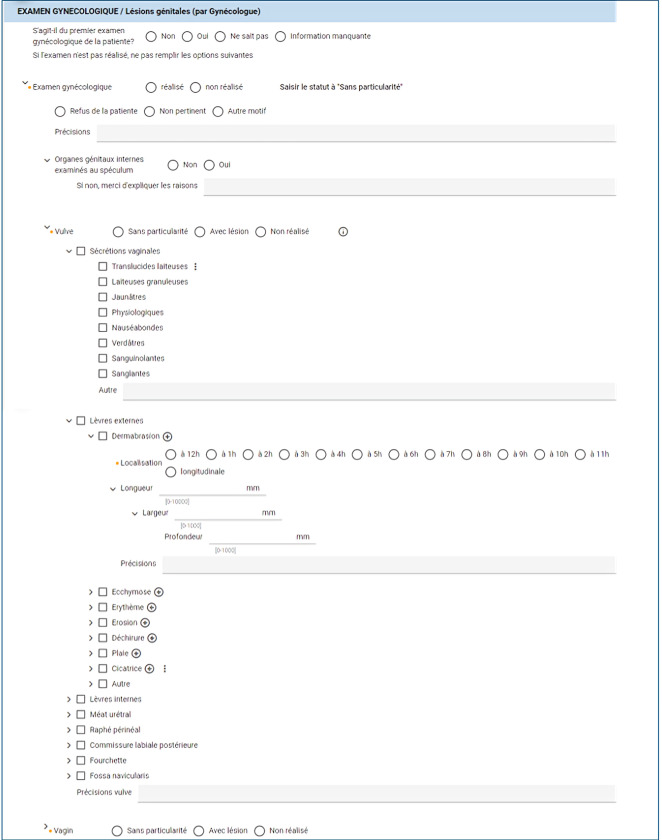
User interface of eSAR (electronic sexual assault records).

A list of eSARs in draft mode (unsigned) as well as some basic patient information can be accessed by authorized personnel (Textbox 1). Doctors receive a notification on their task list when an update or modification is required. This list will help to manage the workflow and help doctors meet their deadlines, (eSARs should be finalized within 30 d). Beyond this deadline, the color of the task in their workflow changes from blue to red and they start to receive reminder notifications. The user interface also allows doctors to click on dedicated buttons to validate sections or complete the report, and simultaneously, a PDF version is automatically generated, ensuring a seamless and efficient way to produce a finalized output (Figures1  2).

Textbox 1. **List of eSARs (electronic sexual assault records) in Draft Mode (unsigned)**

- Finalization of the file (signatures)
- Date of signatures
- Creation of eSAR (date and hour)
- Number of this medical event
- Identification number assigned to the patient
- Name, First name, date of birth (necessary information to identify the file)
- Date of the consultation
- Date de the sexual assault
- Names of the gynecologist and the forensic physician

**Figure 2.**
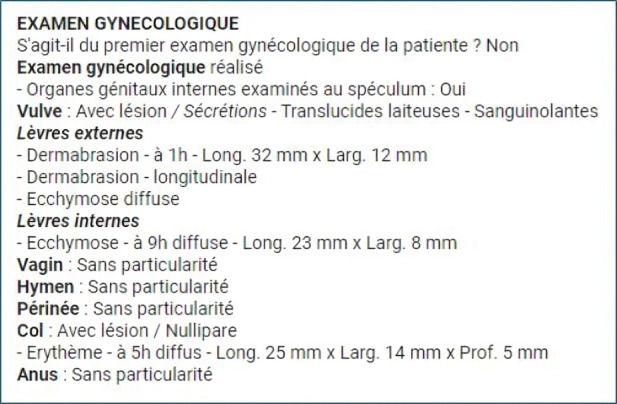
PDF output of eSAR (electronic sexual assault records).

### Interoperability

This electronic sexual assault report form is reproducible in other Swiss cantons or other countries. The eSAR is available in French and translations of all variables are available in English and German. The eSAR is integrated into the EMR platform known as DPI, which has been in use at HUG since 2000. A license is required for the use of DPI, which was acquired by the Valais Hospital in 2023. The informatic languages used are HTML-5 and LOINC. For the use with any other information system other than DPI, the eSAR framework can be exported in CSV file, by contacting the corresponding author of this paper. The eSAR is currently being adapted for patients with penises, both children and adults, in collaboration with the pediatric and adult ED.

### Participating Entities and Budget Planning

The eSAR is a part of a multicentric research project, funded by a grant of 684,029.80 CHF (US $ 752,433) from the Swiss Federal Office for Gender Equality (FOGE) in Switzerland and HUG. The project includes a retrospective study, built on a cohort studied between 2018‐2021 in Geneva and Lausanne. It also includes a prospective study, elaborated to analyze the data collected from eSAR since implementation and a long-term follow-up questionnaire. The FOGE played no role in the design and conduct of the study. FOGE did not fund the development of the eSAR but only the working time of the epidemiologist. The remaining costs were covered by HUG (70,510 CHF; equivalent to US $ 77,561), including creation of the forms (23,000 CHF; 25,300), informatic development (38,400 CHF, equivalent to US $ 42,240), tablets for schemas and signature from patients and physicians (3,360 CHF, equivalent to US $ 3696), methodical support for data analysis (5,750 CHF, equivalent to US $ 6325). Other costs for the human resources were covered by their routine salaries. The ongoing study results will be published in peer-reviewed journals. There are no conflicts of interests to declare.

### Sustainability

Twice a year, training for the care and treatment of patients of sexual assault and the eSAR is organized by the ObGyn ED. It includes theoretical presentations, use of the eSAR and a simulation of triage, history taking, and treatment of a fictitious patient where gender inclusivity and interpersonal skills must be put to practice. These trainings target forensic doctors, nurses, midwives, and gynecologists, some of whom rotate every 6 months. In total, there are 7 forensic doctors and 11 gynecologists (9 residents and 2 chief residents), 10 midwives and 19 nurses.

## Discussion

### Strengths

The eSAR is the first electronic sexual assault record implemented in Switzerland, representing a major advance in standardized documentation and data collection for sexual assault cases. Developed through a multidisciplinary collaboration and continuously refined for both clinical and research purposes, the eSAR provides a robust, real-time dataset through its integration into the EMR. This integration not only enhances patient care and facilitates research, quality improvement, and monitoring of service utilization, but also supports the development of institutional protocols and serves as a foundation for evidence-based policymaking and national best-practice guidelines in sexual assault care.

Interest in this standardized form from various health departments and hospitals continues to enrich ongoing discussions and contributes to the advancement of best practices. Furthermore, the eSAR enables the creation of a regional sexual assault patient cohort, allows tracking of patient loss to follow-up, and provides opportunities to develop performance indicators and improve clinical governance through real-time surveillance and benchmarking. Despite representing only 1.16% of total gynecology emergency consultations, sexual assault cases involve a significant administrative workload, as records must be reviewed and validated by supervisors. By analyzing each step in the care process, eSAR data can help identify opportunities to improve workflow efficiency. Furthermore, the continuous revisions and improvements led by HUG experts strengthen its role as a comprehensive medicolegal tool for both clinical application and research.

### Lessons Learned

Forensic doctors were initially hesitant about the digitalization of the SAR, as they were accustomed to writing paper reports in a free-form manner, which they viewed as an integral part of their medical practice. Concerns were also raised about the time required to complete the eSAR, which was initially estimated to take about an hour. However, once implemented, the eSAR was eventually positively received, as its design effectively aligned with medical practice needs, demonstrating its adaptability and usefulness. As the Chief Forensic doctor was involved in its development, resident and intern forensic doctors, despite any initial reluctance, were required to use the eSAR for all sexual assault reports. Over time, they began to appreciate its advantages, particularly the time saved and the standardization of key variables, which improved both the consistency and quality of documentation.

### Unintended Consequences

The implementation of the eSAR has further strengthened collaboration between departments bringing together professionals from different fields to exchange expertise, align practices, and enhance coordination across specialties. This interdisciplinary dialogue has helped further optimize the HUG’s overall response to sexual assault cases.

### Similar Intervention

Australia [17] and Finland [18] have demonstrated that a national, standardized and centralized approach to sexual assault support centers, increases access to quality and holistic care, as well as collaboration among different institutional actors. The number of police-reported offenses has increased, as have the criminal procedures in addition to patient satisfaction. Systematically collected data and official statistics support the development of targeted government strategies, something we strive to achieve.

### Limitations

A key limitation of the eSAR is that it exclusively includes patients who present to public hospitals and consent to a joint medico-legal examination, as defined by the HUG protocol. This excludes individuals who do not report the assault, who decline medico-legal procedures, or who seek care outside of the hospital system. As a result, the cohort is not representative of all survivors of sexual assault. Future efforts must address how to identify and include these underrepresented populations to ensure a more comprehensive understanding of postassault care needs.

At present, the eSAR is not yet available for patients with penises (ie, children and adults), but its use will become mandatory in the pediatric and adult emergency departments once the adapted version is implemented, as is already the case in gynecology and obstetrics.

### Conclusion

The implementation of eSAR at HUG marks a significant advancement in improving data collection, care processes, and the development of patient-centered services. Establishing a regional or national standard and ultimately, a sexual violence observatory, as recommended by the Istanbul Convention is an achievable goal, requiring collaboration between cantons and coordination at the national level. The eSAR is a replicable model for both adult and minor individuals of all genders. It is a powerful tool in advancing the objectives outlined by GREVIO, particularly the implementation of standardized care pathways and the development of national standards for forensic data collection.

## Supplementary material

10.2196/66764 Multimedia Appendix 1 Swiss Sexual Violence Standardized Data Set - Gynecological and Obstetric Emergencies Unit - HUG.

10.2196/66764 Checklist 1 i-CHECK-DH checklist

## Abbreviations

CURML: Centre Universitaire Romand de Médecine Légale
DPI: Dossier Patient Intégré
EMR: Electronic Medical Record
eSAR: electronic sexual assault record
FOGE: Federal Office for Gender Equality
GREVIO: Group of Experts on Action against Violence against Women and Domestic Violence
HUG: Geneva University Hospital
ObGyn: Obstetrics and Gynecology
ObGyn ED: Obstetrics and Gynecology Emergency Department
PEP: post-exposure prophylaxis
SAR: sexual assault reports
STI: sexually transmitted infections
UIMPV: Interdisciplinary Unit for Medicine and Prevention of Violence

## References

[1] (2019). Le harcèlement sexuel et les violences sexuelles faites aux femmes sont répandus en suisse. GFS.Bern.

[2] Office fédéral de la statistique violence sexualisée: infractions et personnes lésées. Office fédéral de la statistique.

[3] Group of Experts on Action against Violence against Women and Domestic Violence (GREVIO) (2022). GREVIO’s (baseline) evaluation report (switzerland) on legislative and other measures giving effect to the provisions of the council of europe convention on preventing and combating violence against women and domestic violence (istanbul convention). https://rm.coe.int/grevio-inf-2022-27-eng-final-draft-report-on-switzerland-publication/1680a8fc73.

[4] (2022). Plan d’action national de la suisse en vue de la mise en œuvre la convention d’Istanbul de 2022 à 2026. Bureau fédéral de l’égalité entre femmes et hommes (BFEG).

[5] (2024). Unité interdisciplinaire de médecine et prévention de la violence (UIMPV). Hôpitaux universitaires genève.

[6] (2024). Maladies infectieuses. Hôpitaux Universitaires Genève.

[7] La Harpe R, Burkhardt S, Ricard-Gauthier D, Poncet A, Yaron M, Fracasso T (2019). Factors influencing the filing of complaints, their investigation, and subsequent legal judgment in cases of sexual assault. J Forensic Sci.

[8] Cottler-Casanova S, Lourenço V, Guillot C (2023). Sexual assault reporting: study to improve prevention, information, and care of victims in emergency care settings. https://www.hug.ch/sites/interhug/files/structures/GR-Journaliste/documents/constat-agression-sexuelle-en.pdf.

[9] Kjærulff M, Bonde U, Astrup BS (2019). The significance of the forensic clinical examination on the judicial assessment of rape complaints - developments and trends. Forensic Sci Int.

[10] Perrin Franck C, Babington-Ashaye A, Dietrich D (2023). iCHECK-DH: Guidelines and Checklist for the Reporting on Digital Health Implementations. J Med Internet Res.

[11] (2022). Protocole complet de prise en charge au departement de gynecologie et d’Obstetrique des Hopitaux Universitaires de Geneve des personnes victime d’Agression sexuelle. Hôpitaux Universitaires de Genève.

[12] (2016). Strengthening the medico-legal response to sexual violence. https://iris.who.int/bitstream/handle/10665/197498/WHO_RHR_15.24_eng.pdf?sequence=1.

[13] (2003). Guidelines for medico-legal care of victims of sexual violence. World Health Organization.

[14] United Nations Entity for Gender Equality and the Empowerment of Women (UN Women) and World Health Organization (WHO) Global (2022). Technical guidance: improving the collection and use of administrative data on violence against women.

[15] Post-exposure prophylaxis (PEP): recommendations for PEP. European AIDS Clinical Society (EACS).

[16] (2024). Bioscope de l’Université de genève en collaboration avec RTS découverte. Sexesss: Mon corps sous la loupe.

[17] (2023). Outcomes framework 2023–2032: Under the national plan to end violence against women and children 2022–2032. Department of Social Services, Commonwealth of Australia.

[18] Korjamo R (2021). The background of the clients in seri support centers, their use of support services and the progress of the criminal procedure: an interim report (article in swedish). Valtioneuvosto Statsradet.

