# Supplementary material for: Creation and Implementation of an Electronic Sexual Assault Record at the Geneva University Hospital
Source: JMIR Med Inform. 2025 Nov 20;13:e66764. doi: 10.2196/66764 (PMC12634005; doi:10.2196/66764)
Supplement: Multimedia Appendix 1 [file medinform-v13-e66764-s001.docx]

**Appendix 1 – Swiss Sexual Violence Standardized Data Set - Gynecological and Obstetric Emergencies Unit - HUG**

**
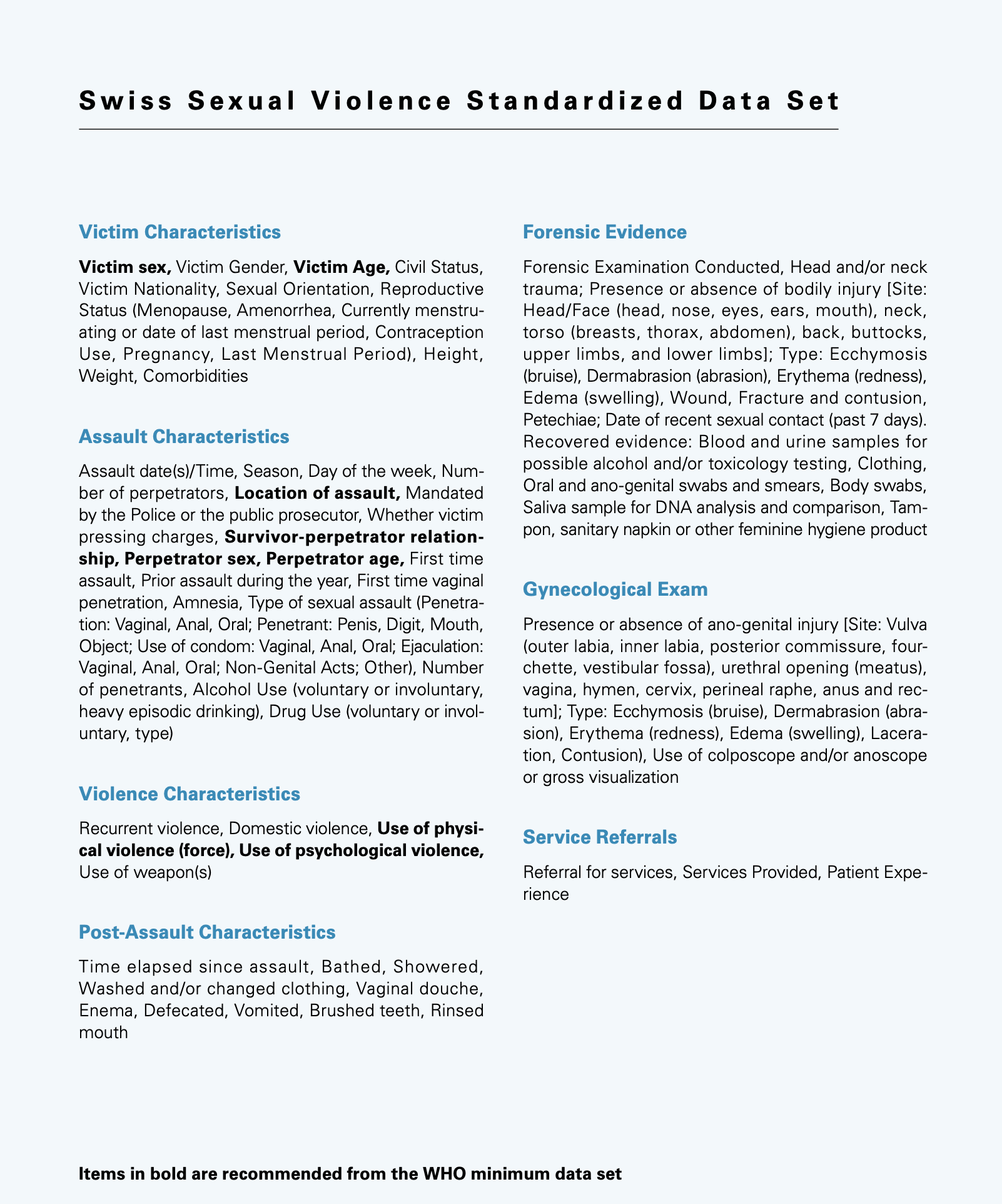
**
